# Tocilizumab Outcomes in Critically Ill COVID-19 Patients Admitted to the ICU and the Role of Non-Tocilizumab COVID-19-Specific Medical Therapeutics

**DOI:** 10.3390/jcm12062301

**Published:** 2023-03-16

**Authors:** Alyaa Elhazmi, Ahmed A. Rabie, Awad Al-Omari, Hani N. Mufti, Hend Sallam, Mohammed S. Alshahrani, Ahmed Mady, Adnan Alghamdi, Ali Altalaq, Mohamed H. Azzam, Anees Sindi, Ayman Kharaba, Zohair A. Al-Aseri, Ghaleb A. Almekhlafi, Wail Tashkandi, Saud A. Alajmi, Fahad Faqihi, Abdulrahman Alharthy, Jaffar A. Al-Tawfiq, Rami Ghazi Melibari, Yaseen M. Arabi

**Affiliations:** 1 Department of Critical Care, Dr. Sulaiman Al-Habib Medical Group, Riyadh 11643, Saudi Arabia; 2 Critical Care Department, King Saud Medical City, Riyadh 11196, Saudi Arabia; 3 Research Center, Dr. Sulaiman Alhabib Medical Group, Riyadh 11643, Saudi Arabia; 4 College of Medicine, Alfaisal University, Riyadh 11533, Saudi Arabia; 5 Section of Cardiac Surgery, Department of Cardiac Sciences, King Faisal Cardiac Center, King Abdulaziz Medical City, MNGHA-WR, Jeddah 21423, Saudi Arabia; 6 College of Medicine, King Saud Bin Abdulaziz University for Health Sciences, Jeddah 11481, Saudi Arabia; 7 Department of Adult Critical Care Medicine, King Faisal Specialist Hospital & Research Centre, Jeddah 23431, Saudi Arabia; 8 Department of Emergency and Critical Care, King Fahad Hospital of the University, Dammam University, Al Khobar 31952, Saudi Arabia; 9 Department of Anesthesiology and Intensive Care, Tanta University Hospital, Tanta 31527, Egypt; 10 Prince Sultan Military Medical City, Military Medical Services, Ministry of Defense, Riyadh 12233, Saudi Arabia; 11 Intensive Care Department, King Abdullah Medical Complex, Jeddah 23816, Saudi Arabia; 12 Department of Medicine, Intensive Care, King Abdulaziz University, Jeddah 21589, Saudi Arabia; 13 Department of Critical Care, King Fahad Hospital, Al Medina Al Munawara 41477, Saudi Arabia; 14 Departments of Emergency Medicine and Critical Care, College of Medicine, King Saud University, Riyadh 11451, Saudi Arabia; 15 College of Medicine, Dar Al Uloom University, Riyadh 13314, Saudi Arabia; 16 Department of Adult Critical Care, Fakeeh Care Group, Jeddah 23323, Saudi Arabia; 17 Department of Surgery, Intensive Care, King Abdulaziz University, Jeddah 21589, Saudi Arabia; 18 Infectious Disease Unit, Specialty Internal Medicine, Johns Hopkins Aramco Healthcare, Dhahran 34464, Saudi Arabia; 19 Infectious Disease Division, Department of Medicine, Indiana University School of Medicine, Indianapolis, IN 46202, USA; 20 Department of Critical Care, King Abdullah Medical City, Makah 24246, Saudi Arabia; 21 Intensive Care Department, King Abdullah International Medical Research Center, College of Medicine, King Saud Bin Abdulaziz University for Health Sciences, Ministry of National Guard Health Affairs, Riyadh 11426, Saudi Arabia

**Keywords:** tocilizumab, COVID-19, outcome, propensity-matching

## Abstract

Background: Tocilizumab is a monoclonal antibody proposed to manage cytokine release syndrome (CRS) associated with severe COVID-19. Previously published reports have shown that tocilizumab may improve the clinical outcomes of critically ill patients admitted to the ICU. However, no precise data about the role of other medical therapeutics concurrently used for COVID-19 on this outcome have been published. Objectives: We aimed to compare the overall outcome of critically ill COVID-19 patients admitted to the ICU who received tocilizumab with the outcome of matched patients who did not receive tocilizumab while controlling for other confounders, including medical therapeutics for critically ill patients admitted to ICUs. Methods: A prospective, observational, multicenter cohort study was conducted among critically ill COVID-19 patients admitted to the ICU of 14 hospitals in Saudi Arabia between 1 March 2020, and October 31, 2020. Propensity-score matching was utilized to compare patients who received tocilizumab to patients who did not. In addition, the log-rank test was used to compare the 28 day hospital survival of patients who received tocilizumab with those who did not. Then, a multivariate logistic regression analysis of the matched groups was performed to evaluate the impact of the remaining concurrent medical therapeutics that could not be excluded via matching 28 day hospital survival rates. The primary outcome measure was patients’ overall 28 day hospital survival, and the secondary outcomes were ICU length of stay and ICU survival to hospital discharge. Results: A total of 1470 unmatched patients were included, of whom 426 received tocilizumab. The total number of propensity-matched patients was 1278. Overall, 28 day hospital survival revealed a significant difference between the unmatched non-tocilizumab group (586; 56.1%) and the tocilizumab group (269; 63.1%) (*p*-value = 0.016), and this difference increased even more in the propensity-matched analysis between the non-tocilizumab group (466.7; 54.6%) and the tocilizumab group (269; 63.1%) (*p*-value = 0.005). The matching model successfully matched the two groups’ common medical therapeutics used to treat COVID-19. Two medical therapeutics remained significantly different, favoring the tocilizumab group. A multivariate logistic regression was performed for the 28 day hospital survival in the propensity-matched patients. It showed that neither steroids (OR: 1.07 (95% CI: 0.75–1.53)) (*p* = 0.697) nor favipiravir (OR: 1.08 (95% CI: 0.61–1.9)) (*p* = 0.799) remained as a predictor for an increase in 28 day survival. Conclusion: The tocilizumab treatment in critically ill COVID-19 patients admitted to the ICU improved the overall 28 day hospital survival, which might not be influenced by the concurrent use of other COVID-19 medical therapeutics, although further research is needed to confirm this.

## 1. Introduction

Tocilizumab is a monoclonal antibody directed against the pro-inflammatory cytokine interleukin 6 (IL-6), resulting in the blockage of IL-6 signaling and reduced inflammatory mediators. It has been proposed to manage cytokine release syndrome (CRS) associated with severe coronavirus disease (COVID-19) after being satisfactorily investigated for treatment-related outcomes, including efficacy [1,2]. Previously published reports have shown that tocilizumab may improve the clinical outcomes of critically ill patients admitted to intensive care units (ICUs). However, although the international Randomized Embedded Multifactorial Adaptive Platform for Community Acquired Pneumonia (REMAP-CAP) trial showed favorable outcomes with IL-6 antagonists [3], the COVACTA trial showed no statistically significant difference in clinical outcomes with tocilizumab [4]. Due to these contradictory findings, precise data about the impact of other medical therapeutics concurrently used to treat COVID-19 alongside tocilizumab are unavailable [5,6,7].

Therefore, we aimed to compare the overall outcome of critically ill COVID-19 patients admitted to the ICU who received tocilizumab with the outcome of matched patients who did not receive tocilizumab while controlling for other confounders, including medical therapeutics for critically ill patients admitted to ICUs, using propensity-score matching and other statistical analysis tools.

## 2. Methods

### 2.1. Study Design

This was a prospective, observational, multicenter cohort study conducted in 14 hospitals in Saudi Arabia. We included critically ill COVID-19 patients admitted to the ICU between 1 March 2020 and 31 October 2020. The data included in this study were obtained from the Saudi COVID-19 platform [8], and institutional review board (IRB) approval was obtained from the Saudi Ministry of Health’s Central IRB on 20 February, 2020, with the code number [20-80E] and from the ethical boards of each participating center. Informed consent was obtained from all subjects and/or their legal guardian(s). Propensity-matching analysis was utilized to compare patients who received tocilizumab to other patients who did not. In addition, we used multivariable logistic regression on the propensity-matched patients to evaluate these therapeutics’ impact on survival. The study adhered to the Standards for Reporting Diagnostic Accuracy Studies (STARD) guidelines (http://www.stard-statement.org/ accessed on 3 February 2023), the Strengthening the Reporting of Observational Studies in Epidemiology (STROBE) guidelines https://www.strobe-statement.org/ accessed on 3 February 2023), and the Helsinki Declaration of 1975.

### 2.2. Settings

The participating ICUs were located in accredited tertiary hospitals. The multidisciplinary treatment team adhered to Saudi Arabia’s Ministry of Health’s (and other internationally published) protocols and guidelines [9]. In addition, during the study period, non-ICU physicians joined the critical care team under the supervision of intensivists after receiving basic ICU management training.

### 2.3. Patients

#### 2.3.1. Inclusion Criteria

1. All critically ill COVID-19 patients admitted to the ICU between 1 March 2020, and 31 October 2020 were included in the study. In each included patient, COVID-19 infection was confirmed by detecting SARS-CoV-2 using real-time polymerase chain reaction (RT-PCR) using nasopharyngeal swabs or tracheal aspirate specimens;
2. All included patients were adults (aged ≥ 18 years).

#### 2.3.2. Exclusion Criteria

1. Patients with a Do-Not-Resuscitate (DNR) order on file;
2. Patients with a diagnosed active, intractable terminal malignancy;
3. Bedbound patients who were diagnosed as being in a vegetative state not conducive to treatment;
4. Patients who were adults <18 years old or children.

### 2.4. Data Collection

The data were collected manually in the CRF and entered into the electronic database Research Electronic Data Capture (REDCap, Vanderbilt University, Nashville, TN, USA) [10]. Next, it was validated using secondary sources. The gathered data included patient demographics, comorbidities, signs and symptoms of COVID-19 illness, laboratory abnormalities, mechanical ventilator (MV) utilization, supplementary therapies, drugs, complications, and outcomes. The arterial oxygen partial pressure to fractional inspired oxygen ratio (PaO_2_:FiO_2_) was calculated for each spontaneously breathing patient by converting O_2_ flow to an estimated FiO_2_ [11]. An immunocompromised state was defined as solid organ malignancy, leukemia, current steroid use (prednisone >7 mg daily for >2 weeks), post-organ transplantation, or rheumatological disease with immunomodulator treatment (such as azathioprine, methotrexate, infliximab, mycophenolate mofetil, or others). Infection was defined as a positive culture in the blood or tracheal aspirate in compliance with the 2018 Declaration of Helsinki.

### 2.5. Outcomes

#### 2.5.1. The Primary Outcome

The overall 28 day hospital survival of patients who received tocilizumab versus matched patients who did not receive tocilizumab.

#### 2.5.2. The Secondary Outcomes

The secondary outcomes include the length of ICU stay and survival to ICU discharge.

## 3. Statistical Analysis

Continuous variables were calculated as medians with interquartile ranges (IQR) of 25–75%, and categorical variables were calculated as frequencies and percentages. Demographics, baseline clinical features, co-interventions, and outcome variables were compared between patients who received tocilizumab in the ICU and those who did not. Student’s t-tests or Wilcoxon rank-sum tests were used for continuous variables. For categorical variables, the chi-square test or the Fisher’s exact test was used. The log-rank test was used to compare the 28 day hospital survival of patients who received tocilizumab with those who did not.

Propensity-score matching was used in a systematic, stepwise manner [11,12]. The initial step was to address some variables’ missing values. Based on Rubin’s taxonomy [13], we assumed that missing variables were missing at random (MAR). Multiple imputations were applied using the mice package in R and the classification and regression trees (CART) method with five runs of imputation and five iterations at each run (a total of 25 complete datasets) [14,15]. A new imputed full dataset was extracted and used to calculate the propensity score using the MatchIt package in R [16,17]. The propensity scores for receiving tocilizumab for all patients in our cohort were calculated, and the “full matching” method results in a ratio of 1:2 (matching one patient receiving tocilizumab to two patients with closely similar characteristics who did not receive tocilizumab), with the following confounders: age in years, body mass index (BMI) in kg/m^2^, gender, diabetes (DM), hypertension (HTN), chronic obstructive pulmonary disease (COPD) or asthma, chronic kidney disease (CKD), need for intubation within the first 48 h from ICU admission, oxygen saturation <90%, C-reactive protein level (CRP), ferritin level, white blood count neutrophil to lymphocyte ratio (WBC N/L) at ICU, sequential organ failure (SOFA) score [8], and PaO_2_:FiO_2_ (Table 1).

The initial analysis of the original dataset was repeated on the propensity-matched dataset (PS dataset). In addition, we used multivariable logistic regression on the propensity-matched patients to evaluate the therapeutics’ impact on survival. All statistical analyses were performed using R software version 4.1.1 (R Foundation for Statistical Computing, Vienna, Austria). All statistical tests were two-sided and deemed significant when the *p*-values were <0.05.

## 4. Results

### 4.1. Patient Characteristics

**Figure 1:**
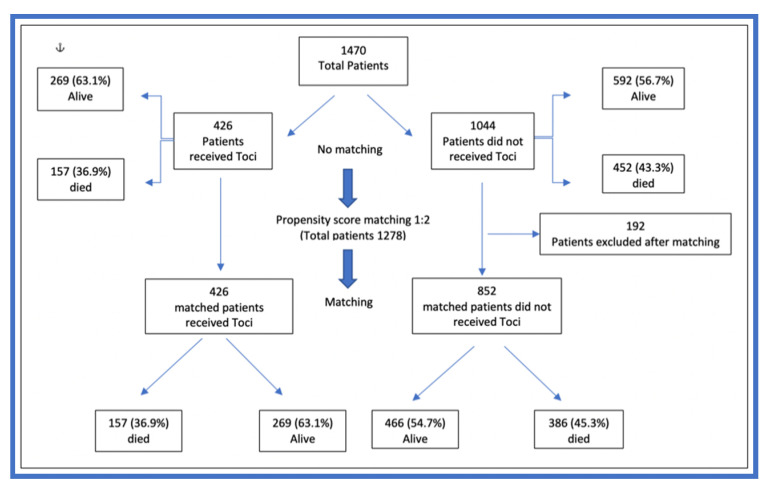
The flowchart of patients’ recruitment and propensity-matching.

Table 1 shows that the total number of unmatched patients was 1470, and 426 patients received tocilizumab. The patients in the tocilizumab group were significantly older than those in the non-tocilizumab group (57 years (IQR 47.5–67) vs. 55 years (IQR 45–66), respectively, *p* = 0.031). The total number of patients with propensity-matching was 1278 (Figure 1). After propensity-matching, age was no longer significantly different between the tocilizumab and non-tocilizumab groups (57 years (IQR 47–67) vs. 56 years (IQR 46–67), respectively, *p* < 0.186).

### 4.2. Medical Therapeutics Combined with Tocilizumab

While the model successfully matched common medical therapeutics seen in COVID-19 between the two groups, two medical therapeutics remained significantly different in favor of the tocilizumab group in the unmatched and propensity-matching analyses. In the non-matched analysis, steroids were given to 694 (66.5%) patients in the non-tocilizumab group and 359 (84.3%) patients in the tocilizumab group (*p* < 0.001), while after propensity matching, they were found to be received by 618 (72.5%) patients in the non-tocilizumab group and 359 (84.3%) patients in the tocilizumab group (*p* = 0.001). Similarly, favipiravir was given to 152 (14.6%) patients in the non-tocilizumab group and 164 (38.5%) patients in the tocilizumab group (*p* < 0.001) in the non-matching analysis, while after the propensity-matching analysis, 136 (16%) patients in the non-tocilizumab group and 164 (38.5%) patients in the tocilizumab group (*p* < 0.001) received it. (Table 1). 

## 5. Outcome

**Figure 2:**
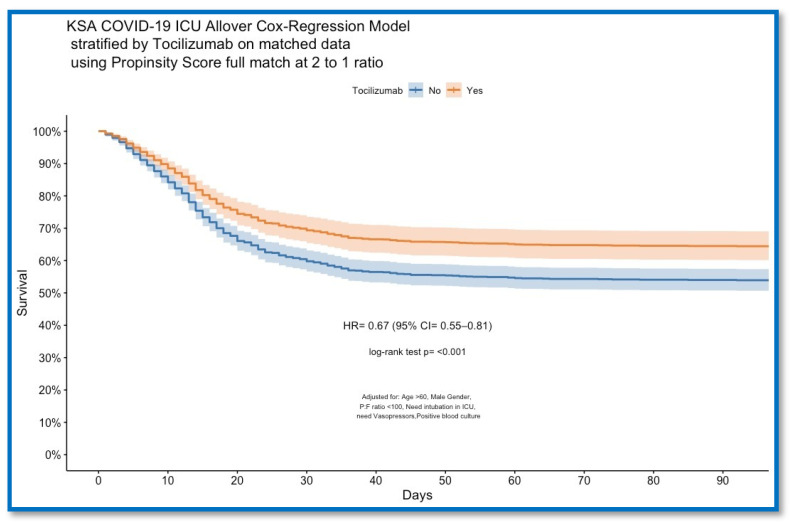
COVID-19 ICU Cox regression model of overall 28 day survival stratified by tocilizumab on propensity-matched data with a 2:1 ratio.

Overall, 28 day hospital survival showed a significant difference in survival between the unmatched non-tocilizumab (586, 56.1%) and tocilizumab (269, 63.1%) groups (*p* = 0.016), and in the propensity-matched analysis between the non-tocilizumab (466, 54.6%) and tocilizumab (269, 63.1%) groups (*p* = 0.005). (Table 2). The log-rank test showed a significant difference in overall 28 day hospital survival with increasing ICU days between the propensity-matched tocilizumab and non-tocilizumab groups (*p* < 0.001), as is shown in Figure 2. Multivariable logistic regression analysis in the full propensity-matched patients that includes tocilizumab as an independent factor showed (OR: 1.38 (95% CI: 0.75–1.53)) (*p* = 0.0332) remained a predictor for 28 day survival and showed also that neither steroids nor favipiravir remained predictors for survival (OR: 1.07 (95% CI: 0.75–1.53)) (*p* = 0.697) and (OR: 1.08 (95% CI: 0.61–1.9)) (*p* = 0.799), respectively, (Table 3).

## 6. Discussion

In this prospective, observational, multicenter cohort study, we found that tocilizumab use increased 28 day survival in critically ill COVID-19 patients; this finding was obtained from analysis of the matching of the dataset (Table 2) and confirmed by multivariable logistic regression (Table 3 and Table S1) and Cox regression (Figure 2). Nevertheless, this study found no effect of medical therapeutics commonly used in COVID-19 on the survival of tocilizumab use.

We used a propensity-score analysis to match the non-tocilizumab and tocilizumab groups to compare outcomes and successfully mitigate the influence of different medical therapeutics on COVID-19 when paired with tocilizumab except for steroids and favipiravir. Thus, we performed a multi-variable logistic regression for overall 28 day hospital survival in the fully propensity-matched patients to eliminate the effects of favipiravir and steroids on the tocilizumab outcome.

Numerous COVID-19 treatments have been evaluated separately for efficacy and have provided inconsistent results for various reasons. There is no consensus regarding their use, as multiple studies conducted in the past have given contradictory results [18,19,20,21,22,23]. Tocilizumab, a recombinant humanized anti-human IL-6 antagonist, is one of these therapeutics that has been extensively investigated, with increasing evidence of its use [22,23]. The drug was introduced in the early 2000s to treat autoimmune disorders such as refractory rheumatoid arthritis and systemic juvenile idiopathic arthritis [24]. Later on, in 2017, it was approved by the FDA for CRS treatment. During the COVID-19 pandemic, the drug significantly improved patient outcomes [25]. A large, randomized trial concluded that tocilizumab is an effective treatment for enhancing the survival outcomes of hospitalized COVID-19 patients with evidence of cytokine release [23]. In their study, Petrak R et al. found that each additional day of delay from admission to tocilizumab administration independently increased the odds of receiving mechanical ventilation by 21% (95% CI: (1.08–1.38), *p* = 0.002) [26]. However, studies have not evaluated combined therapies with tocilizumab. A study conducted by Zhao H et al. found that the combination of an antiviral drug (favipiravir) and an IL-6 receptor blocker (tocilizumab) can significantly reduce mortality in COVID-19 patients [27]. In this trial, we searched for other therapeutics that may contribute to the outcomes of tocilizumab patients. Next to steroids, favipiravir was the second therapeutic that may be associated with the outcomes of tocilizumab patients. Nevertheless, the multiple logistic regression did not retain this effect (OR = 2.46).

Mikulska et al. was an early study that provided a hint of the favorable outcome of the combined therapy of steroids with tocilizumab, in which the methylprednisolone + tocilizumab treatment arm demonstrated a superior outcome in averting death and intubation compared to single therapy arms [28]. A recently published systematic review and meta-analysis by Lim et al. showed that the concurrent use of a corticosteroid such as methylprednisolone was a significant influencer of tocilizumab efficacy in reducing mortality and improving survival [29]. This is especially visible in the excellent outcomes demonstrated by the CHIC study, which used 3–6 days of methylprednisolone in combination with tocilizumab in the late stages of the disease [30]. RECOVERY [23] and REMAP-CAP [3] have also confirmed the efficacy of combined therapy with corticosteroids and tocilizumab. Unlike previously mentioned cohorts, corticosteroids had no role in tocilizumab increasing 28 day survival in our cohort; this could be because the patients received corticosteroids in the late stage of the disease when they were already very sick.

More than three years after the first cases of COVID-19 were reported, multiple treatment options have been investigated for short-term outcomes; evidence gaps exist for long-term outcomes and quality of life. The REMAP-CAP trial investigators most recently closed these gaps by evaluating six treatment classes for 4689 patients admitted to the intensive care unit with COVID-19 from March 2020 through June 2021 for long-term outcomes (180 days) and quality of life. One of the main findings is that IL-6 receptor antagonists like tocilizumab again showed a very high >99.9% posterior probability of superiority over a placebo (adjusted hazard ratio, 0.74 [95% credible interval CrI, 0.61–0.90]). In addition, this 180 day outcome provides reassurance that the early mortality benefit from IL-6 receptor antagonists did not result in longer-term adverse outcomes like late opportunistic infection and others [31].

The strength of the study is the prospective and multicenter design of the trial, which provides a relatively big cohort to be evaluated. In addition, the research was conducted according to a registered protocol with a propensity score matching design.

### Limitations

The findings of the current study should be interpreted while considering its limitations. Firstly, prospective observational studies cannot draw cause-and-effect inferences due to known and unknown confounders. Secondly, misclassifying the data is possible as the data were collected from electronic health records in some hospitals and manually in others. Third, this cohort enrolled patients at a relatively early stage of the pandemic, when there were only a few small variations in the usual clinical management of COVID-19 patients in general and the indications for tocilizumab in particular that were independent of interleukin 6 levels, leaving confounders unaccounted for. Furthermore, the median time of tocilizumab administration was not collected, but it was administered within 48 h of ICU admission. Finally, our study focused on Saudi Arabian patients. However, the country’s population is diverse, and only 14 hospitals were included in the study, which may limit its applicability to other geographical areas.

## 7. Conclusions

The tocilizumab treatment in critically ill COVID-19 patients admitted to the ICU improved the overall 28 day hospital survival rate with favorable outcomes on the length of ICU stay and the survival to ICU discharge that might not be influenced by the concurrent use of other COVID-19 medical therapeutics, although further research is needed to confirm this.

## Figures and Tables

**Table 1:** Characteristics, demographics, and combined interventions of tocilizumab and non-tocilizumab patients.

Variable	Unmatched Patients (*n* = 1470)					Propensity-Score-Matched Patients (*n* = 1278)				
	Overall (*n* = 1470)	No Tocilizumab (*n* = 1044)	Tocilizumab (*n* = 426)	*p*-Value	SMD Unadjusted	Overall (*n* = 1278)	No Tocilizumab (*n* = 852)	Tocilizumab (*n* = 426)	*p*-Value	SMD Adjusted
Age (years) median (IQR)	56 (45–66)	55 (45–66)	57 (47.5–67)	0.031	0.105	56 (46–67)	56 (45–67)	57 (47–67)	0.186	0.052
Gender male, *n* (%)	382 (26.0)	249 (23.9)	133 (31.2)	0.008	−0.068	336 (26.3)	203 (23.8)	133 (31.2)	0.006	−0.561
	1085 (73.8)	792 (75.9)	293 (68.8)			942 (73.7)	649 (76.2)	293 (68.8)		
BMI, *n* (%) <30	761 (51.8)	571 (54.7)	191 (44.8)	<0.001	0.293	686 (53.7)	495 (58.1)	191 (44.8)	<0.001	0.168
>30	608 (41.4)	374 (35.8)	235 (55.2)			592 (46.3)	357 (41.9)	235 (55.2)		
DM, *n* (%)	770 (52.4)	531 (50.9)	244 (57.3)	0.002	0.048	695 (54.4)	451 (52.9)	244 (57.3)	0.159	0.008
HTN, *n* (%)	676 (46.0)	457 (43.8)	225 (52.8)	<0.001	0.058	627 (49.1)	402 (47.2)	225 (52.8)	0.066	0.009
IHD, *n* (%)	184 (12.5)	125 (12.0)	64 (13.8)	<0.001		183 (14.3)	119 (14.0)	64 (15.0)	0.672	
CKD, *n* (%)	123 (8.4)	76 (7.3)	47 (11.0)	0.432	0.047	117 (9.1)	72 (8.4)	45 (10.5)	0.551	0.007
BA or COPD, *n* (%)	149 (10.1)	84 (8.0)	66 (15.5)	<0.001	0.061	152 (11.9)	86 (10.1)	66 (15.5)	0.007	0.048
Immune def, *n* (%)	72 (4.9)	27 (2.6)	45 (10.6)	<0.001		72 (5.6)	27 (3.2)	45 (10.6)	<0.001	
Admitted to the hospital already intubated, *n* (%)	128 (8.7)	98 (9.4)	30 (7.0)	0.167		109 (8.5)	79 (9.3)	30 (7.0)	0.215	
SOFA, median (IQR)	5 (3–8)	5 (2–8)	5 (3–7)	0.144	−0.125	5 (3–8)	5 (2–8)	5 (3–7)	0.2	−0.049
ROX, median (IQR)	4 (3–6)	4 (3–5)	4 (3–6)	0.422		4 (3–6)	4 (3–7)	4 (3–6)	0.532	
Mean arterial pressure, *n* (%) <65	135 (9.2)	92 (8.8)	43 (10.1)	0.241		126 (9.9)	83 (9.7)	43 (10.1)	0.921	
>65 (mmHg)	1296 (88.2)	920 (88.1)	383 (89.9)			1152 (90.1)	769 (90.3)	383 (89.9)		
Need for vasopressors in the first 5 days of ICU, *n* (%)	395 (26.9)	288 (27.6)	107 (25.1)	0.366		355 (27.8)	248 (29.1)	107 (25.1)	0.151	
PO2:FiO2 ratio (mmHg) (ICU admission) median (IQR)	101 (70.5–163)	105 (72.2–171)	94.8 (66.9–138)	<0.001	−0.610	97.5 (68.4–148)	98.6 (69.1–151)	94.8 (66.9–138)	0.265	−0.054
Intubation within the first 48 hrs of ICU admission *n*%	594 (40.4)	443 (42.4)	151 (35.4)	0.016	−0.069	509 (39.8)	358 (42.0)	151 (35.4)	0.028	−0.075
Laboratory data on ICU admission: WBC (10^9^/L), mean (±SD)	10.66 (6.29)	11.03 (6.04)	9.83 (6.77)	0.001		10.65 (6.29)	11.04 (6.02)	9.83 (6.77)	0.002	
WBC NL ratio, mean (±SD)	10.30 (8.68)	10.17 (8.33)	10.47 (9.14)	0.465	0.012	10.44 (8.64)	10.43 (8.38)	10.47 (9.14)	0.937	−0.076
Creatinine (mmol), mean (±SD)	139.58 (166.59)	149.16 (180.7)	118.69 (130.27)	0.002		134.89 (157.1)	143.22 (169.47)	118.69 (130.27)	0.007	
CRP (mg/L), mean (±SD)	121.82 (98.78)	118.18 (100.9)	125.65 (93.31)	0.17	0.048	124.20 (99.25)	123.47 (102.13)	125.65 (93.31)	0.712	0.059
D-dimer (mg/L), mean (±SD)	1.50 (1.07)	1.58 (1.12)	1.88 (1.15)	0.005		1.77 (1.14)	1.54 (1.07)	1.88 (1.15)	<0.001	
Ferritin (mcg/L), mean (±SD)	1006 (781.10)	985.64 (788.61)	1012.91 (775.00)	0.362	−0.007	1052.1(802.5)	1071.70 (815.68)	1012.91 (775.00)	0.217	−0.047
IMV, *n* (%)	778 (52.9)	562 (53.8)	217 (50.9)	0.302		677 (52.9)	460 (54.0)	217 (50.9)	0.294	
ECMO, *n* (%)	71 (4.8)	43 (4.1)	28 (6.6)	0.116		66 (5.2)	38 (4.5)	28 (6.6)	0.14	
Steroids, *n* (%)	1085 (73.8)	694 (66.5)	359 (84.3)	<0.001		977 (76.4)	618 (72.5)	359 (84.3)	0.001	
Convalescent plasma, *n* (%)	53 (3.6)	7 (0.7)	46 (10.8)	<0.001		54 (4.2)	7 (0.7)	46 (10.8)	<0.001	
Chloroquine, *n* (%)	429 (29.2)	288 (27.6)	141 (33.1)	0.001		384 (30.0)	241 (28.3)	141 (33.1)	0.061	
Favipiravir, *n* (%)	316 (21.5)	152 (14.6)	164 (38.5)	<0.001		302 (23.6)	136 (16.0)	164 (38.5)	<0.001	
Ramdesivir, *n* (%)	13 (0.9)	7 (0.7)	6 (1.4)	0.001		12 (0.9)	6 (0.7)	6 (1.4)	0.356	
Ribavirin, *n* (%)	241 (16.4)	160 (15.3)	83 (19.5)	0.001		219 (17.1)	136 (16.0)	83 (19.5)	0.135	
Interferon, *n* (%)	152 (10.3)	97 (9.3)	55 (12.9)	0.001		141 (11.0)	85 (10.0)	55 (12.9)	0.107	
IVIG, *n* (%)	51 (3.5)	39 (3.7)	12 (2.8)	0.001		46 (3.6)	34 (4.0)	12 (2.8)	0.367	

SMD: standard mean difference; BMI: Body mass index; DM: Diabetes mellitus; HTN: Hypertension; IHD: Ischemic heart disease (def: known based on a coronary angiogram, cardiac CT, non-invasive diagnosis, or previous clinical diagnosis); CKD: Chronic kidney disease (def: GFR <60 mL/min per 1.73 m^2^); BA: Bronchial asthma; COPD: Chronic obstructive pulmonary disease, immunocompromised: (def: solid organ malignancy, leukemia, on steroids (prednisone >7 mg daily for >two weeks), post organ transplant at any time); SOFA: Sequential organ failure; ROX: Respiratory rate-oxygenation; PO_2_/FiO_2_: Partial oxygen pressure to fraction inspired oxygen ratio; WBC: White blood cells; ECMO: Extracorporeal membrane oxygenation; CRP: C-reactive protein; IMV: Invasive Mechanical Ventilation; and IVIG: Immunoglobulin therapy.

**Table 2:** Complications and patient outcomes.

Variable	Unmatched Patients (*n* = 1044)				Propensity-Score-Matched Patients (*n* = 1278)			
	Overall (*n* = 1470)	No Tocilizumab (*n* = 1044)	Tocilizumab (*n* = 426)	*p*-Value	Overall (*n* = 1278)	No Tocilizumab (*n* = 852)	Tocilizumab (*n* = 426)	*p*-Value
ICU length of stay, median (IQR)	9 (5–16)	8 (4–15)	12 (7–21)	<0.001	10 (5–17)	9 (4–15)	12 (7–21)	<0.001
Hospital length of stay, median (IQR)	15 (9–24)	14 (8–23)	18 (12–30)	<0.001	15 (10–25)	15 (9–23)	18 (12–30)	<0.001
MV duration (days) median (IQR)	7 (0–14)	6 (0–13)	8 (0–15)	0.538	3 (0–12)	3 (0–11)	3 (0–13)	0.288
DVT, *n* (%)	33 (2.2)	20 (1.9)	14 (3.3)	0.304	31(2.4)	17 (2.0)	14 (3.3)	0.222
PE, *n* (%)	44 (3.0)	33 (3.2)	11 (2.6)	0.241	39(3.1)	28 (3.3)	11 (2.6)	0.605
Pneumothorax, *n* (%)	89 (6.1)	57 (5.5)	32 (7.5)	0.114	84(6.6)	52 (6.1)	32 (7.5)	0.402
MI, *n* (%)	64 (4.4)	50 (4.8)	16 (3.8)	0.255	52(4.1)	36 (4.2)	16 (3.8)	0.802
Cardiac arrest, *n* (%)	379(25.8)	280 (26.8)	99 (23.2)	0.267	332 (26.0)	232 (27.2)	100 (23.5)	0.169
RRT, *n* (%)	227(15.4)	148 (14.2)	79 (18.5)	0.058	202 (15.8)	123 (14.4)	79 (18.5)	0.069
Stroke, *n* (%)	32 (2.2)	23 (2.2)	9 (2.1)	0.431	29 (2.3)	20 (2.3)	9 (2.1)	0.947
ICH, *n* (%);	33 (2.2)	23 (2.2)	10 (2.3)	0.986	31 (2.4)	21 (2.5)	10 (2.3)	1
ICU survival to discharge, *n* (%)	868 (59.0)	592 (56.7)	276 (64.8)	0.005	748 (58.5)	472 (55.4)	276 (64.8)	0.002
28 day hospital overall survival, *n* (%)	855 (58.2)	586 (56.1)	269 (63.1)	0.016	735 (57.5)	466 (54.7)	269 (63.1)	0.005

MV: Mechanical ventilation; DVT: Deep venous thrombosis; PE: Pulmonary embolism; MI: myocardial infarction; RRT: Renal replacement therapy; and ICH: intracerebral hemorrhage.

**Table 3:** Multivariate logistic regression for the overall 28 day hospital survival in the fully propensity-matched patients.

						Univariate					Multivariate			
			Variable		Units	Odds Ratio	CI.95	*p*-Value			Odds Ratio	CI.95	*p*-Value	
1	Age	>60	Ref						Ref					
					<60	1.57	[1.26;1.97]	<1 × 10^−4^		S	1.63	[1.21;2.19]	0.001175	S
2	Gender	Male	Ref						Ref					
					Female	1	[0.78;1.28]	0.9919		NS	1.8	[0.82;3.96]	0.141482	NS
3			DM		Yes	Ref					Ref			
					No	1.25	[1.00;1.57]	0.04802		S	0.9	[0.64;1.24]	0.510627	NS
4	HTN	Yes	Ref						Ref					
					No	1.48	[1.18;1.85]	0.000616		S	1.56	[1.12;2.18]	0.008437	S
5	COPD	Yes	Ref						Ref					
					No	0.8	[0.57;1.12]	0.1905		NS	0.73	[0.48;1.11]	0.136765	NS
6	CKD	Yes	Ref						Ref					
					No	1.28	[0.89;1.85]	0.1822		NS	0.92	[0.59;1.44]	0.710813	NS
7	PaO_2_:FiO_2_.	<100	Ref											
		>100	1.34	[1.07;1.68]		0.01072		S	1.41	[1.07;1.85]		0.01503	S	
8	IMV	Yes	Ref						Ref					
		No	9.94	[7.59;13.02]		<1 × 10^−4^		S	7.05	[5.22;9.54]		<1 × 10^−4^	S	
9	Vasopressors	Yes	Ref						Ref					
		No	4.99	[3.81;6.52]		<1 × 10^−4^		S	2.68	[1.97;3.65]		<1 × 10^−4^	S	
10	Tocilizumab	No	Ref						Ref					
		Yes	1.36	[1.07;1.73]		0.01164		S	1.38	[1.03;1.85]		0.033253	S	
11	Steroids	Yes	Ref						Ref					
		No	1.32	[0.99;1.75]		0.05849		NS	1.07	[0.75;1.53]		0.697434	NS	
12	Favipiravir	Yes	Ref						Ref					
		No	0.99	(0.78–1.28)		0.99		NS	1.08	(0.61–1.9)		0.799	NS	
13	WBC.NLratio	>8.5	Ref						Ref					
		≤8.5	1.45	[1.16;1.81]		0.001215		S	1.08	[0.82;1.43]		0.573611	NS	
14	Ferritin	>1400	Ref						Ref					
		≤1400	1.48	[1.16;1.89]		0.001493		S	1.41	[1.04;1.91]		0.025717	S	
15	Ddimer	>1.5	Ref						Ref					
		≤1.5	1.81	[1.45;2.27]		<1 × 10^−4^		S	1.45	[1.10;1.91]		0.008396	S	
16	CRP	>150	Ref						Ref					
		≤150	1.12	[0.89;1.40]		0.3541		NS	0.86	[0.64;1.14]		0.286094	NS	
		No	2.47	[1.92;3.17]		<1 × 10^−4^		S	1.38	[1.02;1.87]		0.036575	S	

DM: Diabetes mellitus; HTN: Hypertension; CKD: Chronic kidney disease (def: GFR <60 mL/min per 1.73 m^2^); COPD: Chronic obstructive pulmonary disease; PO_2_/FiO_2_: Partial oxygen pressure to fraction inspired oxygen ratio; IMV: Invasive Mechanical Ventilation; WBC: White blood cells; and CRP: C-reactive protein.

## Data Availability

The datasets used and analyzed during the current study are available from the corresponding author upon reasonable request.

## Supplementary Materials

The following supporting information can be downloaded at: https://www.mdpi.com/article/10.3390/jcm12062301/s1, Table S1: Multivariable logistic regression analysis that includes all variables with significant differences after matching and included tocilizumab as a dependent factor.

## References

[1] Campochiaro C., Della-Torre E., Cavalli G., De Luca G., Ripa M., Boffini N., Tomelleri A., Baldissera E., Rovere-Querini P., Ruggeri A. (2020). Efficacy and safety of tocilizumab in severe COVID-19 patients: A single-center retrospective cohort study. Eur. J. Intern. Med..

[2] Canziani L.M., Trovati S., Brunetta E., Testa A., De Santis M., Bombardieri EGuidelli G., Albano G., Folci M., Squadroni M., Beretta G.D. (2020). Interleukin-6 receptor blocking with intra-venous Tocilizumab in COVID-19 severe acute respiratory distress syndrome: A retrospective case-control survival analysis of 128 patients. J. Autoimmune.

[3] The Remap-Cap Investigators (2021). Interleukin-6 Receptor Antagonists in Critically Ill Patients with COVID-19. N. Engl. J. Med..

[4] Rosas I.O., Brau N., Waters M., Go R.C., Hunter B.D., Bhagani S., Skiest D., Aziz M.S., Cooper N., Douglas I.S. (2021). Tocilizumab in Hospitalized Patients with Severe COVID-19 Pneumonia. N. Engl. J. Med..

[5] Stone J.H., Frigault M.J., Serling-Boyd N.J., Fernandes A.D., Harvey L., Foulkes A.S., Horick N.K., Healy B.C., Shah R., Bensaci A.M. (2020). Efficacy of Tocilizumab in patients hospitalized with COVID-19. N. Engl. J. Med..

[6] Salvarani C., Dolci G., Massari M., Merlo D.F., Cavuto S., Savoldi L., Bruzzi P., Boni F., Braglia L., Turrà C. (2021). Effect of Tocilizumab vs. standard care on clinical worsening in patients hospitalized with COVID-19 Pneumonia: A randomized clinical trial. JAMA Intern. Med..

[7] Hermine O., Mariette X., Tharaux P.L., Resche-Rigon M., Porcher R., Ravaud P. (2021). Effect of Tocilizumab vs. usual care in adults hospitalized with COVID-19 and moderate or severe Pneumonia: A randomized clinical trial. JAMA Intern. Med..

[8] Elhazmi A., Al-Omari A., Sallam H., Mufti H.N., Rabie A.A., Alshahrani M., Mady A., Alghamdi A., Altalaq A., Azzam M.H. (2022). Machine learning decision tree algorithm role for predicting mortality in critically ill adult COVID-19 patients admitted to the ICU. J. Infect. Public Health.

[9] National Center for Disease Control. https://ncdc.gov.in/index1.php?lang=1&level=1&sublinkid=703&lid=550..

[10] Read K., LaPolla F.W.Z. (2018). A new hat for librarians: Providing REDCap support to establish the library as a central data hub. J. Med. Libr. Assoc..

[11] Vincent J.L., Moreno R., Takala J., Willatts S., De Mendonça A., Bruining H., Reinhart C.K., Suter P., Thijs L.G. (1996). The SOFA (Sepsis-related Organ Failure Assessment) score to describe organ dysfunction/failure. On behalf of the Working Group on Sepsis-Related Problems of the European Society of Intensive Care Medicine. Intensive Care Med..

[12] Austin P. (2011). A tutorial and case study in propensity score analysis. An application to estimating the effect of in-hospital smoking cessation counseling on mortality. Multivar. Behav Res..

[13] Harris H., Horst S.J. (2016). A brief guide to decisions at each step of the propensity score matching process. Pract. Assess. Res. Eval..

[14] Leyrat C., Seaman S.R., White I.R., Douglas I., Smeeth L., Kim J., Resche-Rigon M., Carpenter J.R., Williamson E.J. (2019). Propensity score analysis with partially observed covariates: How should multiple imputations be used?. Stat. Methods Med. Res..

[15] Choi J., Dekkers O.M., le Cessie S. (2019). A comparison of different methods to handle missing data in the context of propensity score analysis. Eur. J. Epidemiol..

[16] Olmos A., Govindasamy P. (2015). Propensity scores: A practical introduction using R. J. Multidiscip. Eval..

[17] Zhang Z., Kim H.J., Lonjon G., Zhu Y. (2019). Balance diagnostics after propensity score matching. Ann. Transl. Med..

[18] Alkofide H., Almohaizeie A., Almuhaini S., Alotaibi B., Alkharfy K.M. (2021). Tocilizumab and Systemic Corticosteroids in the Management of Patients with COVID-19: A Systematic Review and Meta-Analysis. Int. J. Infect. Dis..

[19] Berardicurti O., Ruscitti P., Ursini F., D’Andrea S., Ciaffi J., Meliconi R., Iagnocco A., Cipriani P., Giacomelli R. (2020). Mortality in tocilizumab-treated patients with COVID-19: A systematic review and meta-analysis. Clin. Exp. Rheumatol..

[20] Malgie J., Schoones J.W., Pijls B.G. (2021). Decreased Mortality in Coronavirus Disease 2019 Patients Treated with Tocilizumab: A Rapid Systematic Review and Meta-analysis of Observational Studies. Clin. Infect. Dis..

[21] Lan S.H., Lai C.C., Huang H.T., Chang S.P., Lu L.C., Hsueh P.R. (2020). Tocilizumab for severe COVID-19: A systematic review and meta-analysis. Int. J. Antimicrob. Agents.

[22] Tleyjeh I.M., Kashour Z., Damlaj M., Riaz M., Tlayjeh H., Altannir M., Altannir Y., MAl-Tannir M., Tleyjeh R., Hassett L. (2021). Efficacy and safety of tocilizumab in COVID-19 patients: A living systematic review and meta-analysis. Clin. Microbiol. Infect..

[23] RECOVERY Collaborative Group (2021). Tocilizumab in patients admitted to hospital with COVID-19 (RECOVERY): A randomized, controlled, open-label, platform trial. Lancet.

[24] De Benedetti F., Brunner H.I., Ruperto N., Kenwright A., Wright S., Calvo I., Cuttica R., Ravelli A., Schneider R., Woo P. (2012). Randomized trial of tocilizumab in systemic juvenile idiopathic arthritis. N. Engl. J. Med..

[25] Roche FDA Approves Roche’s Actemra/RoActemra (Tocilizumab) for Treating CAR T Cell-Induced Cytokine Release Syndrome. https://www.roche.com/media/releases/med-cor-2017-08-30.htm.htm.

[26] Petrak R.M., Skorodin N.C., Van Hise N.W., Fliegelman R.M., Pinsky J., Didwania V., Anderson M., Diaz M., Shah K., Chundi V.V. (2021). Tocilizumab as a Therapeutic Agent for Critically Ill Patients Infected with SARS-CoV-2. Clin. Transl. Sci..

[27] Zhao H., Zhu C., Zhang C., Jiawen Li Wei M., Qin Y., Zhao H., Zhu Q., Zhang C., Li J., Wei M. (2021). Tocilizumab combined with favipiravir in the treatment of COVID-19: A multicenter trial in a small sample size. Biomed. Pharmacother..

[28] Mikulska M., Nicolini L.A., Signori A., Biagio A.D., Sepulcri C., Russo C., Dettori S., Berruti M., Sormani M.P., Giacobbe D.R. (2020). Tocilizumab and steroid treatment in patients with COVID-19 Pneumonia. PLoS ONE.

[29] Lim P.C., Wong K.L., Rajah R., Chong M.F., Chow T.S., Subramaniam S., Lee C.Y. (2022). Comparing the efficacy of tocilizumab with corticosteroid therapy in treating COVID-19 patients: A systematic review and meta-analysis. DARU J. Pharm. Sci..

[30] Ramiro S., Mostard R.L.M., Magro-Checa C., van Dongen C.M.P., Dormans T., Buijs J., Gronenschild M., De Kruif M.D., Van Haren E.H., Van Kraaij T. (2020). Historically controlled comparison of glucocorticoids with or without tocilizumab versus supportive care only in patients with COVID-19-associated cytokine storm syndrome: Results of the CHIC study. Ann. Rheum. Dis..

[31] Writing Committee for the REMAP-CAP Investigators (2023). Long-term (180-Day) Outcomes in Critically Ill Patients With COVID-19 in the REMAP-CAP Randomized. Clin. Trial. JAMA.

