# Supplementary material for: Tocilizumab Outcomes in Critically Ill COVID-19 Patients Admitted to the ICU and the Role of Non-Tocilizumab COVID-19-Specific Medical Therapeutics
Source: J Clin Med. 2023 Mar 16;12(6):2301. doi: 10.3390/jcm12062301 (PMC10053430; doi:10.3390/jcm12062301)
Supplement: Supplementary file 1 [file jcm-12-02301-s001.zip › jcm-2214177-supplementary.pdf]

Supplementary

Suppl. Table S1:

Multivariable logistic regression analysis that includes all variables with significant differences after matching and included tocilizumab as a dependent factor.

			Multivariate		
	**Variable**	**Units**	**OddsRatio**	**CI.95**	**p-value**
**1**	Demo.Age.Years.Cat60	60+	Ref		
		<60	1.63	[1.21;2.19]	0.001175
**2**	Demo.Gender	Male	Ref		
		Female	1.8	[0.82;3.96]	0.141482
**3**	Comorb.DM	Yes	Ref		
		No	0.9	[0.64;1.24]	0.510627
**4**	Comorb.HTN	Yes	Ref		
		No	1.56	[1.12;2.18]	0.008437
**5**	Comorb.BAorCOPD	Yes	Ref		
		No	0.73	[0.48;1.11]	0.136765
**6**	Comorb.CKD	Yes	Ref		
		No	0.92	[0.59;1.44]	0.710813
**7**	ICU0.PFratio.All.O2.Delivary.Cat100	Ref			
		100+	1.41	[1.07;1.85]	0.01503
**8**	ICUstay.need.ETT	Yes	Ref		
		No	7.05	[5.22;9.54]	< 1e-04
**9**	ICU.1to5.Ino	Yes	Ref		
		No	2.68	[1.97;3.65]	< 1e-04
**10**	ICUmeds.TocilizumabV2	No	Ref		
		Yes	1.38	[1.03;1.85]	0.033253
**11**	Steroids	Yes	Ref		
		No	1.07	[0.75;1.53]	0.697434
**12**	LABSicu0.WBC.NLratio.Cat	>8.5	Ref		
		<=8.5	1.08	[0.82;1.43]	0.573611
**13**	LABSicu0.FerritinV2.Cat	>1400	Ref		
		<=1400	1.41	[1.04;1.91]	0.025717
**14**	LABSicu0.Ddimer.Cat	>1.5	Ref		
		<=1.5	1.45	[1.10;1.91]	0.008396
**15**	LABSicu0.CRPV2.Cat	>150	Ref		
		<=150	0.86	[0.64;1.14]	0.286094
**16**	CULT.blood.Positive	Yes	Ref		
		No	1.82	[1.32;2.50]	0.000246
**17**	CULT.resp.Positive	Yes	Ref		
		No	1.38	[1.02;1.87]	0.036575
